# Workplace relational injustice and authoritarian treatment as risk factors for sexual harassment at a large Swedish university: results from a cross-sectional study

**DOI:** 10.1186/s12889-025-25226-2

**Published:** 2025-11-06

**Authors:** Frida Pilgaard, Anette Agardh, Per-Olof Östergren, Jack Palmieri, Benedict Oppong Asamoah

**Affiliations:** Division of Social Medicine and Global Health, Department of Clinical Sciences, Malmö, Clinical Research Centre, Lund University, Malmö, 202 13 Sweden

**Keywords:** Sexual harassment, Authoritarian treatment, Relational justice, Academia, Organisational climate, Unfair treatment

## Abstract

**Background:**

Workplace sexual harassment remains a persistent issue in Sweden. Research about organisational antecedents has been criticised for being limited in scope, possibly overlooking contextual factors relevant for the occurrence of sexual harassment. This study examined two factors capturing aspects of organisational climate, perceived relational justice and authoritarian treatment, and their association to workplace sexual harassment in a Swedish university setting.

**Methods:**

Cross-sectional data from a 2019 survey targeting all Lund University employees was used. Validated instruments were employed to measure relational justice, authoritarian treatment and sexual harassment. Bivariate and multivariable regression analyses, stratified by gender, were conducted using Poisson regression models and additive interaction analysis performed, calculating synergy index (SI) and relative excess risk due to interaction (RERI).

**Results:**

The adjusted prevalence ratio (PR) of sexual harassment was statistically significantly higher among participants who perceived low relational justice (RJ), with a PR of 1.8 (95% CI 1.3–2.6) among women and PR 2.2 (95% CI 1.01–4.6) among men. A similar pattern was seen among participants who perceived high authoritarian treatment (AT), with a PR of 2.0 (95% CI 1.4–2.9) among women and PR 3.4 (95% CI 1.7–6.9) among men. Interaction analyses indicated that women and participants with a foreign background perceiving low relational justice or high authoritarian treatment have a higher probability of sexual harassment compared to men and participants with a Swedish background with the same perceptions. Furthermore, that participants with low or non-academic positions perceiving low relational justice have a lower probability of sexual harassment compared to those with high academic positions.

**Conclusion:**

Our findings suggest that superiors’ unjust or authoritarian treatment of employees might be contextual factors contributing to workplace sexual harassment, disproportionally affecting women, employees with a foreign background or high academic positions. To ensure evidence-based interventions, researchers should incorporate organisational risk factors in the design and evaluation of workplace interventions aimed at reducing sexual harassing behaviours.

## Background

Workplace sexual harassment remains a persistent global concern [1, 2]. Data from a recent EU wide survey shows that one in three women in the EU have experienced sexual harassment at work in their lifetime [3]. Academia is no exception [4], and at Lund University, one of the largest universities in Sweden, 25% of female employees and 7% of male employees report that they have, at some point during their employment, experienced at least one of ten behaviours defined as sexual harassment [5]. Women are the main targets of sexual harassment [2, 6], especially younger women [3, 7] and women in positions of power [6, 8, 9], but also women marginalised by, for example, sexual orientation or race [6, 10] and women in precarious employment [7, 11].

Sexual harassment has been a subject of research for almost half a century [7]. Yet, within the research community, a universally accepted definition of the phenomenon is still lacking, and conceptual clarity has been called for [12]. In this study, we base our understanding of sexual harassment on the definition provided in Swedish law, focusing exclusively on unwanted behaviours with a sexual connotation [13].

Negative consequences of sexual harassment are reported at both the individual and organisational levels [2] including poor mental health outcomes [14, 15], absence due to sickness [16], organisational withdrawal, decreased job commitment [2] and costs for organisations [7]. Individual and organisational antecedents of sexual harassment have also been identified. There is evidence indicating that men with a high likelihood of sexually harassing women cognitively link social dominance with sexuality [17], and that men holding sexist attitudes [18] or having a strong identification with being “males” [19] are more prone to sexually harass. However, the likelihood of an individual with the proclivity to harass engaging in such behaviour depends greatly on the context [18]. Organisational characteristics related to gender balance, workplace climate and culture have been convincingly linked to the occurrence of sexual harassment in organisations [2, 20]. For example, in organisations where men outnumber women and the job tasks are thought to be stereotypically masculine in nature [21], sexual harassment levels are generally higher. Furthermore, organisations tolerating sexual harassment without consequences [22] or having a masculinity contest culture, i.e. workplace cultures embracing masculine norms such as showing no weakness and putting work first [23], are linked to higher levels of sexual harassment. However, research about organisational antecedents has been criticised as having too narrow a perspective, mainly focusing on gender-related aspects [24]. Organisational responses to combat sexual harassment have been guided by this narrow focus, resulting in efforts that mainly produce policies, training and reporting systems [2, 24]. These attempts have not been effective enough in shifting the trajectory of workplace SH [2]. Recently, the concept of organisational justice climate has been introduced in sexual harassment research as a potentially relevant predictor of its occurrence, as well as a factor that may moderate the impact of gender imbalance and perceived tolerance of sexual harassment [24]. These findings are relevant, as they broaden the understanding of which organisational-level interventions might be effective in reducing these undesired workplace behaviours.

Our study seeks to contribute to this emerging research field by examining two factors that capture aspects of organisational climate, perceived relational justice and authoritarian treatment, and their relation to the occurrence of workplace sexual harassment in a Swedish university setting. These aspects have been rarely studied in relation to sexual harassment. We examine individual perceptions of treatment by superiors and management, based on the notion that superiors in an organisation have powerful normative influence on employees, shaping local social norms about which behaviours are accepted and not [25–28]. Relational or interactional justice constitutes an aspect of organisational justice, reflecting whether employees perceive their treatment by superiors as respectful and appropriate including the provision of sufficient and adequate information [29, 30]. Cross-sectional studies show associations between perceived injustice and counterproductive work behaviours such as organisational aggression, anti-social behaviours and bullying [31]. Other studies examining associations indicate that workplace injustice influences attitudes and behaviours of entire work groups [32, 33]. Authoritarian treatment refers to employees’ perceptions of being treated in an authoritarian manner, mainly by their superiors, and constitutes a social dimension (vulnerability) of precarious employment as defined by the precarious employment scale EPRES [34]. Authoritarian leadership has been suggested to contribute to a climate of workplace harassment and bullying [35]. Such leaders tend to avoid open communication with employees and prefer a top-down approach when making decisions [36]. Moreover, an authoritarian leadership style can create a climate of fear [37], discouraging any objections to the treatment of oneself or others, resulting in a culture of silence. Universities are often characterised by hierarchical structures, and it seems plausible that such structures may facilitate authoritarian leadership styles. Hence, the aim of this study is to investigate whether employees’ perceptions of relational justice and authoritarian treatment, respectively, are associated with the occurrence of sexual harassment at a large university in Sweden. We hypothesise that prevalence of sexual harassment will be higher among both women and men who perceive either relational justice (RJ) to be low or authoritarian treatment (AT) to be high. Further, to explore possible variation in sexual harassment risk among subgroups, we investigate to what extent potential interaction between the perceived climate (RJ and AT) and the variables of gender, age, background (Swedish/foreign), type of employment (permanent/temporary) and academic position (high/low or other) influence sexual harassment risk. We hypothesise that women, younger participants, participants with a foreign background, temporary employment or a low or non-academic position will be more affected by an unjust or authoritarian working climate in terms of experiences of sexual harassment, compared to men, older participants, participants with a Swedish background, permanent employment or high academic positions.

## Methods

### Data collection and study population

For this study we used data from a cross-sectional survey conducted at Lund University as a part of a larger project named “Tellus”. Initiated in 2018, this project aimed to strengthen the preventive work against sexual harassment at the university [38]. The survey was sent out in November 2019, in both English and Swedish, by email to all employees (*N* = 8,238), including full time PhD students who have employment status at Swedish universities. To ensure anonymity, responses could not be linked to individual email addresses. The survey remained open for nine weeks, during which two reminders were sent out by email, and the final response rate was 33% (*n* = 2,750). Participants with missing data on both sex and gender, age, and those who did not answer any of the 10 questions on experiences of sexual harassment, altogether 14 individuals, were excluded from the study population yielding a final study population of 2,736 individuals. Basic characteristics were compared between the survey participants and the target population, with minor differences observed in terms of gender and age. Women were slightly over-represented and both male and female participants marginally older than the overall target population. Furthermore, there was a slight overrepresentation of employees with permanent employment compared to those with temporary employment. Professional group affiliations were largely similar, which is noteworthy given that the diversity of occupations among university staff may lead to differences in classification between university administrative records and self-report. A detailed comparison between study participants and the target population including the overall results from the survey [5] and details about the survey instrument [39] have been published elsewhere.

### Outcome variable

As an outcome variable we used sexual harassment during the last 12 months at Lund University. The instrument used to measure sexual harassment was the ‘Lund University Sexual Harassment Inventory’ (LUSHI), developed and validated within the Tellus project [39]. LUSHI comprises the following list of ten behaviours, including one representing sexual violence: unwelcome suggestive looks or gestures; unwelcome soliciting or pressuring for ‘dates’; unwelcome ‘inadvertent’ brushing or touching; unwelcome bodily contact such as grabbing or fondling; unwelcome gifts; unwelcome comments; unwelcome contact by post or telephone; unwelcome contact online, for example, via social media or email; stalking; and attempts to conduct, or the conduct of, oral, vaginal, or anal sex, or other equivalent sexual activity in which one did not participate voluntarily. The study participants were asked if they had experienced any of these behaviours in connection with their employment at Lund University with the answer options: Yes, once; Yes, more than once; and No. All participants who answered yes to at least one of the above behaviours and stated that the behaviour/s occurred during the past 12 months were classified as sexually harassed at the workplace during the last 12 months.

### Exposure variables

Two exposure variables, workplace relational justice and authoritarian treatment, were investigated in this study. To measure relational justice, we used a slightly modified version of the construct developed within the Whitehall II study [40] (i.e. minor adjustments were made to the phrasing of the questions). The construct included the following five items: unfair treatment by supervisor/manager, clarity of information from supervisor/manager, adequate information from supervisor/manager, supervisor/manager willingness to listen to problems, receiving credit for work. Responses for each item were coded on a 0–3 scale, and mean scores were calculated for each participant, ranging between 0 and 15. The tertile value for the overall population was used as a cut-off to divide participants into groups perceiving the relational justice to be high (value range 0–3) or low (value range 4–15). Internal consistency was assessed with Cronbach’s alpha, yielding a result of 0.82.

Authoritarian treatment was measured by four items assessing worry about demanding better conditions, fear of being fired for not complying with employer, authoritarian treatment, feeling replaceable. Responses for each item were coded on a 0–3 scale, and mean scores were calculated for each participant, ranging between 0 and 12. The tertile value for the overall population was used as a cut-off to divide participants into groups perceiving authoritarian treatment to be low (value range 0–3) or high (value range 4–12). Internal consistency was assessed with Cronbach’s alpha, yielding a result of 0.77. The response options for both exposure variables ranged from never to often, referring to participants’ general experiences in their current workplace as no specific time frame was provided.

### Covariates

The survey included two questions related to gender: “What gender were you assigned at birth?” (female/male) and “What is your current gender identity?” (female/male/I do not identify as male or female). Participants were classified as women, men, or non-binary based on their response to the second question. If data for this question was missing (*n* = 15), the response to the first question was used for categorisation. To obtain information about age the participants were asked to indicate which of the following age groups they belonged to; 30 years or younger, 31–40 years, 41–49 years, 50–59 years, or 60 years or older. Foreign background was categorised according to the definition used by Statistics Sweden [41], whereby participants who are either born abroad or have two parents born abroad are classified as having a foreign background. Participants with missing information about their parents’ country of birth were presumed to have a Swedish background if they were born in Sweden (*n* = 5). Professional position was initially specified according to nine types in the survey, which were, for the purpose of analysis, aggregated into the following six categories: professors, senior lecturers, lecturers and researchers, PhD students, administrative and technical support staff, and others. Additional information about type of employment was obtained by participants indicating whether their employment was permanent or temporary.

### Statistical analysis

Preliminary analyses, including cross-tabulations and chi-square tests, were conducted to explore associations between the variables. Bivariate and multivariable regression analyses, stratified by gender, were conducted using Poisson regression models with robust variances [42], using Stata version 18. Results are presented as prevalence ratios (PR) with 95% confidence intervals (CI). The bivariate analyses assessed associations between each covariate and the outcome variable sexual harassment, separately. The multivariable analyses examined the associations between the exposure variables (relational justice and authoritarian treatment) and the outcome variable sexual harassment, adjusting for potential confounders. We added potential confounders stepwise in two different models. The choice of potential confounders was informed by the initial descriptive and bivariate analyses as well as prior research identifying factors linked to our outcome variable and considered potentially relevant to the perception of relational justice and authoritarian treatment in the workplace. Following the multivariable regression modelling in Stata, the post command margins [43] was used to calculate the fully adjusted predictions of the prevalence of sexual harassment across different groups of the independent variables to facilitate interpretation of the result.

To explore potential variation in the risk of sexual harassment across subgroups, we conducted interaction analyses using the reri command in Stata. This procedure generates estimates of additive interaction, including synergy index (SI) and relative excess risk due to interaction (RERI), along with corresponding confidence intervals [44–46]. Unadjusted ORs were calculated for dummy variables combining the perception of relational justice and authoritarian treatment respectively with gender, age (≤ 40 years vs. 40 + years), background (Swedish vs. foreign), type of employment (permanent vs. temporary) and academic position (“high” vs. “low or other”). We based the academic position on participants’ professional positions, categorising Professors and Senior lecturers as having “high” academic positions, and the remaining participants were categorised together in one group as “low or other” academic position. The same calculations were made using ORs adjusted for age, as a sensitivity analysis.

Missing values were treated as missing and reported for all variables in Table 1.

**Table 1:** Characteristics of the study population including experiences of sexual harassment (SH), perceived relational justice and authoritarian treatment , by gender. University staff & PhD students at Lund University, *N* = 2736. Missing reported for all variables where it occurs

	Women (*n* = 1551)		Men (*n* = 1161)		Non-binary (*n* = 24)		Total (*N* = 2736)	
	n	%	n	%	n	%	n	%
Age group								
≤ 30	188	12.1	144	12.4	3	12.5	335	12.2
31–40	373	24.1	250	21.5	11	45.8	634	23.2
41–49	467	30.1	300	25.8	5	20.8	772	28.2
50–59	365	23.5	320	27.6	2	8.3	687	25.1
≥ 60	158	10.2	147	12.7	3	12.5	308	11.3
Professional position								
Professors	81	5.2	203	17.5	2	8.3	286	10.5
Senior Lecturers	189	12.2	193	16.6	3	12.5	385	14.1
Lecturers and Researchers	243	15.7	227	19.6	5	20.8	475	17.4
PhD Student	223	14.4	170	14.6	5	20.8	398	14.6
Admin & Technical	758	48.9	334	28.8	7	29.2	1,099	40.2
Other	56	3.6	33	2.8	2	8.3	91	3.3
*Missing*	*1*	*0.1*	*1*	*0.1*	*0*	*0*	*2*	*0.1*
Type of employment								
Permanent	1,111	71.6	817	70.4	15	62.5	1,943	71.0
Temporary	420	27.1	314	27.1	9	37.5	743	27.2
*Missing*	*20*	*1.3*	*30*	*2.6*	*0*	*0*	*50*	*1.8*
Background								
Swedish	1,183	76.3	868	74.8	15	62.5	2,066	75.5
Foreign	368	23.7	291	25.1	9	37.5	668	24.4
*Missing*	*0*	*0*	*2*	*0.2*	*0*	*0*	*2*	*0.1*
Relational justice								
High	941	60.7	766	66.0	12	50.0	1,719	62.8
Low	567	36.6	355	30.6	11	45.8	933	34.1
*Missing*	*43*	*2.8*	*40*	*3.5*	*1*	*4.2*	*84*	*3.1*
Authoritarian treatment								
Low	890	57.4	783	67.4	8	33.3	1,681	61.4
High	615	39.7	348	30.0	16	66.7	979	35.8
*Missing*	*46*	*3.0*	*30*	*2.6*	*0*	*0*	*76*	*2.8*
SH								
No	1,432	92.3	1,127	97.1	21	87.5	2580	94.3
Yes	119	7.7	34	2.9	3	12.5	156	5.7

### Ethical considerations

The study followed the principles outlined in the 1964 Helsinki Declaration and its subsequent amendments and was approved by the Regional Ethical Review Board in Lund (Dnr 2018/350).

## Results

In all, 2,736 individuals participated in the study, among whom 57% were women, 42% men and 1% of non-binary gender (Table 1). No major differences were found regarding sociodemographic characteristics between men and women, except men generally having higher positions compared to women. Perceptions of low relational justice (RJ) and high authoritarian treatment (AT) were more common among women (36.6% low RJ, 39.7% high AT) compared to men (30.6% low RJ, 30.0% high AT), as were experiences of sexual harassment (7.7% among women, 2.9% among men) (Table 1). The largest proportions of participants reporting experiences of sexual harassment, low relational justice and high authoritarian treatment were found among non-binary gender participants. Table 1 shows the characteristics of the study population, including perceived relational justice, authoritarian treatment and experiences of sexual harassment, by gender. Non-binary gender participants were excluded from further analysis due to small numbers (24 individuals).

Among women and men who perceived either low relational justice (RJ) or high authoritarian treatment (AT) (*n* = 1,302), 42% reported both low RJ and high AT, 31% only high AT, and 27% only low RJ (data not shown).

### Bivariate regression analyses result

Among women, the bivariate regression analysis revealed an approximately twofold higher prevalence of sexual harassment in the youngest age group (≤ 30 years) compared to the oldest (≥ 60 years) (Table 2). No other statistically significant associations were found between any of the other sociodemographic variables examined and sexual harassment. Regarding perceptions of working climate, the bivariate analysis showed that, among both women and men, a low level of relational justice and a high level of authoritarian treatment were associated with an approximately twofold higher prevalence of sexual harassment compared to those who perceived these aspects of the work climate more positively (Table 2).

**Table 2:** Unadjusted association between background variables, perceived relational justice , authoritarian treatment and sexual harassment (SH) at Lund university during the last 12 months, by gender. Bivariate regression analysis result presented as prevalence ratios (PR) with 95% confidence interval (CI). University staff & PhD students at Lund University, *N* = 2736. Missing reported in parenthesis

Independent variables (missing)	Women *n* = 1551			Men *n* = 1161		
	cases/exposed	PR	95% CI	cases/exposed	PR	95% CI
Age groups						
≤ 30	24/188	2.2	1.1–4.7	4/144	1.0	0.3–4.0.3.0
31–40	34/373	1.6	0.8–3.3	7/250	1.0	0.3–3.5
41–49	32/467	1.2	0.6–2.5	12/300	1.5	0,5-4.5.5
50–59	20/365	1.0	0.4–2.1	7/320	0.8	0.2–2.7
≥ 60	9/158	1 (ref)		4/147	1 (ref)	
Professional position (*n* = 2)						
Professors	10/81	1 (ref)		7/203	1 (ref)	
Senior Lecturers	18/189	0.8	0.4–1.6	6/193	0.9	0.3–2.6
Lecturers/Researchers	17/243	0.6	0.3–1.2	7/227	0.9	0.3–2.5
PhD Students	17/223	0.6	0.3–1.3	3/170	0.5	0.1–1.9
Admin/Tech staff	53/758	0.6	0.3–1.1	10/334	0.9	0.3–2.2
Other	4/56	0.6	0.2–1.7	1/33	0.9	0.1–6.9
Employment form (*n* = 50)						
Permanent	83/1111	1 (ref)		22/817	1 (ref)	
Temporary	34/420	1.1	0.7–1.6	9/314	1.1	0.5–2.3
Background (*n* = 2)						
Swedish	86/1183	1 (ref)		26/868	1 (ref)	
Foreign	33/368	1.2	0.8–1.8	8/291	0.9	0.4–2.0.4.0
Relational justice (*n* = 84)						
High	57/941	1 (ref)		16/766	1 (ref)	
Low	60/567	1.7	1.2–2.5	15/355	2.0	1.01–4.0.01.0
Authoritarian treatment (*n* = 76)						
Low	47/890	1 (ref)		15/783	1 (ref)	
High	66/615	2.0	1.4–2.9	18/348	2.7	1.4–5.3

### Association between relational justice and authoritarian treatment and sexual harassment after adjusting for potential confounders

After adjustment for age, background (Swedish/foreign), professional position and type of employment, a low level of relational justice was associated with an approximately twofold higher prevalence of sexual harassment, with a prevalence ratio (PR) of 1.8 (95% CI 1.3–2.6) among women and PR 2.2 (95% CI 1.01–4.6) among men (Table 2). A similar pattern was seen among participants who perceived high level of authoritarian treatment, with a PR of 2.0 (95% CI 1.4–2.9) among women and PR 3.4 (95% CI 1.7–6.9) among men (Table 3).

**Table 3:** Adjusted prevalence ratios (PR) for workplace sexual harassment (SH) during the last 12 months at Lund University, in relation to the perception of relational justice and authoritarian treatment at the workplace, by gender. University staff & PhD students at Lund University, *N* = 2736, non-binary gender excluded

		Women			Men	
		Model 1^a^	Model 2^b^		Model 1^a^	Model 2^b^
	Cases/ exposed	PR (95% CI)	PR (95% CI)	Cases/ exposed	PR (95% CI)	PR (95% CI)
Relational justice						
High	57/941	1.0 (ref)	1.0 (ref)	16/766	1.0 (ref)	1.0 (ref)
Low	60/567	1.8 (1.3–2.5)	1.8 (1.3–2.6)	15/355	2.0 (0.98–4.1)	2.2 (1.01–4.6)
Authoritarian treatment						
High	66/615	1.9 (1.3–2.8)	2.0 (1.4–2.9)	18/348	2.7 (1.4–5.4)	3.4 (1.7–6.9)
Low	47/890	1.0 (ref)	1.0 (ref)	15/783	1.0 (ref)	1.0 (ref)

^a^ Model 1: Adjusted for age

^b^ Model 2: Adjusted for age, background, professional position and type of employment

### Estimated prevalence of sexual harassment by levels of relational justice and authoritarian treatment

**Fig. 1:**
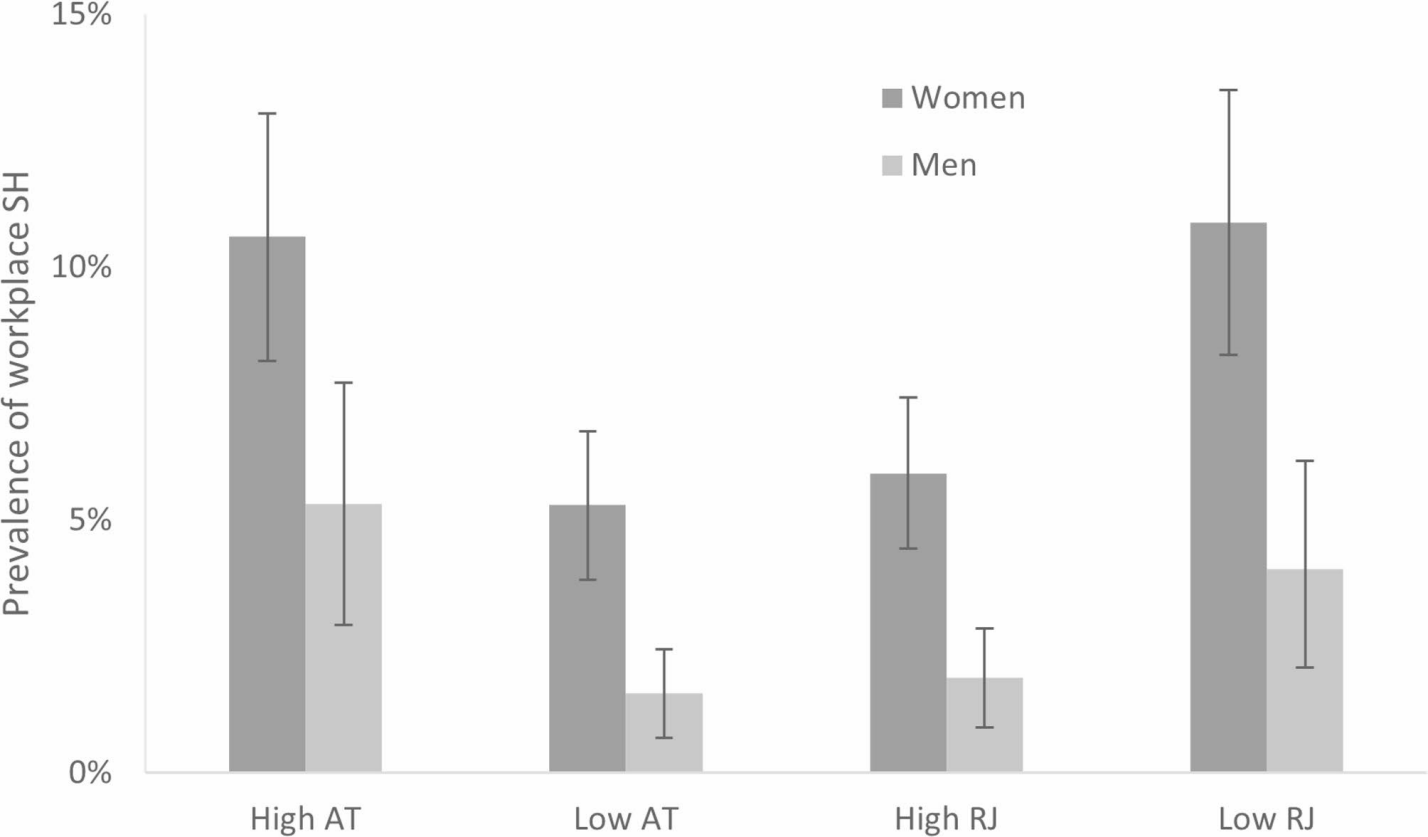
Estimated prevalence of workplace sexual harassment (SH) during the last 12 months by gender and perceived authoritarianism (AT) and relational justice (RJ). Differences are statistically significant at the 5% level

The predicted adjusted prevalence of sexual harassment (SH) by levels of relational justice (RJ) and authoritarian treatment (AT) for women were as follows; low RJ 10.9% SH (95% CI 8.2–13.5), and high RJ 5.9% SH (95% CI 4.4–7.4), high AT 10.6% SH (95% CI 8.1–12.0), low AT 5.3% SH (95% CI 3.8–6.8). The corresponding results for men were: low RJ 4.0% SH (95% CI 1.9–6.2), high RJ 1.9% SH (95% CI 0.9–2.9), high AT 5.3% SH (95% CI 2.9–7.7), low AT 1.6% SH (95% CI 0.7–2.4) (Fig. 1).

### Interaction between relational justice, authoritarian treatment and background characteristics regarding probability of sexual harassment

Interaction analysis results indicated that women perceiving low relational justice or high authoritarian treatment had an increased probability of sexual harassment compared to men with the same perception of relational justice (RJ) and authoritarian treatment (AT) (SI/RERI values 1.45/1.47 (RJ) and 1.41/1.51 (AT)) (Table 4). Similarly, participants with a foreign background perceiving low relational justice or high authoritarian treatment had an increased probability of sexual harassment compared to participants with a Swedish background with the same perception of relational justice or authoritarian treatment (SI/RERI values 2.05/0.88 (RJ) and 3.01/1.25 (AT)) (Table 4). We found indication of antagonistic interaction between relational justice and academic position such that participants with low or non-academic positions perceiving low relational justice had lower probability of SH compared to participants with high academic positions with the same perception of relational justice (SI/RERI 0.55/−0.74) (Table 4). All Confidence intervals included the null, indicating statistical imprecision.

**Table 4:** Interaction result, performed by using dummy variables combining background characteristics with the perception of relational justice and authoritarian treatment . Unadjusted odds ratios (OR) with 95% confidence interval (CI) presented for the outcome sexual harassment (SH) during the last 12 months and the synergy index (SI) and the relative excess risk due to interaction (RERI) result. University staff & PhD students at Lund University, *N* = 2736, non-binary gender excluded

	Relational justice			Authoritarian treatment	
	SH Yes/no	OR (CI 95%)		SH Yes/no	OR (CI 95%)
Gender					
Man + RJH	16/750	1 (ref)	Man + ATL	15/768	1 (ref)
Man + RJL	15/340	2.07 (1.01–4.2)	Man + ATH	18/330	2.79 (1.4–5.6)
Woman + RJH	57/884	3.02 (1.7–5.3)	Woman + ATL	47/843	2.85 (1.6–5.2)
Woman + RJL	60/507	5.55 (3.2–9.7)	Woman + ATH	66/549	6.16 (3.5–10.9)
	SI: 1.47 (95% CI 0.8–2.6) RERI: 1.45 (95% CI −0.6-3.5)			SI: 1.41 (95% CI 0.8–2.5) RERI: 1.51 (95% CI −0.7-3.7)	
Age					
Old + RJH	37/1062	1 (ref)	Old + ATL	35/1100	1 (ref)
Old + RJL	44/558	2.26 (1.4–3.6)	Old + ATH	43/528	2.56 (1.6–4.1)
Young + RJH	36/572	1.81 (1.1–2.9)	Young + ATL	27/511	1.66 (0.99–2.8)
Young + RJL	31/289	3.08 (1.9–5.1)	Young + ATH	41/351	3.67 (2.3–5.9)
	SI: 1.00 (95% CI 0.5–1.9) RERI: 0.01 (95% CI −1.5-1.5)			SI: 1.20 (95% CI 0.6–2.1) RERI: 0.45 (95% CI −1.1-2.0)	
Background					
Swe + RJH	54/1218	1 (ref)	Swe + ATL	53/1255	1 (ref)
Swe + RJL	53/664	1.80 (1.2–2.7)	Swe + ATH	54/632	2.02 (1.4–3.0.4.0)
Foreign + RJH	19/415	1.03 (1.03–1.8)	Foreign + ATL	9/355	0.60 (0.3–1.2)
Foreign + RJL	22/183	2.71 (1.6–4.6)	Foreign + ATH	30/247	2.9 (1.8–4.6)
	SI: 2.05 (95% CI 0.6–6.8) RERI: 0.88 (95% CI −0.5-2.3)			SI: 3.01 (95% CI 0.7–12.8) RERI: 1.25 (95% CI −0.4-2.5)	
Academic position					
“High” + RJH	17/395	1 (ref)	“High” + ATL	21/428	1 (ref)
“High” + RJL	23/207	2.58 (1.3–4.9)	“High” + ATH	18/176	2.08 (1.1–4.0.1.0)
“Low” + RJH	56/1238	1.05 (0.6–1.8)	“Low” + ATL	41/1183	0.71 (0.4–1.2)
“Low” + RJL	52/639	1.89 (1.08–3.3)	“Low” + ATH	66/701	1.92 (1.2–3.2)
	SI: 0.55 (95% CI 0.2–1.4) RERI: −0.74 (95% CI −2.3-0.8)			SI: 1.16 (95% CI 0.2–5.4) RERI: 0.13 (95% CI −1.1-1.3)	
Type of employment					
Perm + RJH	52/1178	1 (ref)	Perm + ATL	48/1214	1 (ref)
Perm + RJL	51/603	1.92 (1.3–2.9)	Perm + ATH	51/573	2.25 (1.5–3.4)
Temp + RJH	20/437	1.04 (0.6–1.8)	Temp + ATL	12/373	0.81 (0.4–1.5)
Temp + RJL	23/241	2.16 (1.3–3.6)	Temp + ATH	33/308	2.71 (1.7–4.3)
	SI: 1.17 (95% CI 0.4–3.8) RERI: 0.17 (95% CI −1.0-1.4)			SI: 1.57 (95% CI 0.6–4.3) RERI: 0.62 (95% CI −0.7-1.9)	

*RJH* Relational justice high, *RJL* Relational justice low, *ATL* Authoritarian treatment low, *ATH* Authoritarian treatment high, *Swe* Swedish, *Perm* Permanent, *Temp* Temporary

We also found indications of a synergetic interaction between authoritarian treatment and type of employment; however, this result was not stable in the sensitivity analysis. All other interaction analyses results were stable in our sensitivity analysis using ORs adjusted for age.

## Discussion

In this study, associations between employees perceived treatment by superiors, in terms of relational justice and authoritarian treatment, and workplace sexual harassment are examined. This expands existing knowledge regarding contextual factors that might contribute to sexual harassing behaviours at workplaces. As hypothesised, after controlling for relevant confounders, a perceived low level of relational justice or high level of authoritarian treatment were associated with an approximately twofold higher prevalence of workplace sexual harassment, among both women and men. Our results indicate that respectful and fair treatment of employees by superiors and management play a role in relation to the occurrence of workplace sexual harassment. This interpretation of our results is supported by other research findings. For example, it has been demonstrated that men who feel unfairly and disrespectfully treated by their supervisors report a greater likelihood of engaging in sexually harassing behaviours [47], which could partly explain the increased levels of sexual harassment among participants perceiving a more unjust and authoritarian leadership in our study. Furthermore, it has been suggested that perceived injustice may function as a job stressor contributing to counter productive work behaviours [31]. Such counter productive work behaviours may include sexual harassment. Also, if the management is perceived as unjust, it is plausible that employees interpret their own, or colleagues, unjust behaviours, such as harassment, to be “ok” according to the local norms, triggering individuals with the proclivity to harass to engage in such behaviour. Perceived authoritarian treatment may contribute to a workplace climate marked by fear and silence, where harassment is less likely to be reported and thus allowed to persist. Our findings are relevant as they broaden the understanding of contextual factors that may contribute to, and facilitate, sexual harassment in the workplace, and generate important questions regarding implications for prevention.

Most SH research has been limited in scope, often treating sexual harassment as an isolated phenomenon [48] and proposing countermeasures that focus primarily on sexual harassment alone, approaches that have proven insufficient [2]. However, sexual harassment rarely occurs in isolation but seems to exist within a larger context of disrespect and mistreatment. For example, Lim and Cortina [49] show that sexual harassment most often co-occurs with incivility, general hostility or other mistreatment, and among employees at Lund University experiences of sexual harassment correlates with other types of harassment as well as derogatory treatment [50]. Perhaps, as sexual harassment does not occur in isolation, countermeasures should likewise not be limited to targeting sexual harassment alone. This broader prevention perspective has been proposed by scholars [24, 51], suggesting promotion of a more general climate of fair and respectful treatment, in combination with non-tolerance of harassing behaviours, as a general anti-harassment (including sexual harassment) counteraction strategy. This more general approach, focusing on justice and respect, may not only help mitigate sexual harassment, but also reduce identity-based mistreatment more broadly, for example, based on ethnicity or sexual orientation [51]. It may further benefit other organisational outcomes, such as work productivity, employee commitment, and perceived work-related stress, all of which have been linked to organisational justice [29]. Moreover, promoting fairness and respect in general, in combination with not tolerating any type of harassment, may be a more widely accepted and feasible approach than interventions targeting sexual harassment alone, as a just and respectful treatment is a concern of all employees.

In line with our second hypothesis, the results of the interaction analysis suggest that women and foreign-born employees are more negatively affected by a perceived unjust or authoritarian work climate, as reflected in a higher probability of experiencing sexual harassment. This increased sexual harassment probability can be understood in the light of intersectional theories [52] emphasising that social identities, such as gender and ethnicity, are associated with varying levels of privilege and power, which may influence individuals’ vulnerabilities in different contexts, for example in a context of a perceived unjust or authoritarian work climate. Contrary to our hypothesis, however, we found indications that employees with a high academic position are more negatively affected by perceived low relational justice in terms of probability of sexual harassment compared to employees with a low or non-academic position. Although not hypothesised, and seemingly counterintuitive, other studies confirm that women in positions of power face higher risk of experiencing sexual harassment [9]. It is widely acknowledged in the literature that sexual harassment is primarily driven by power and dominance rather than sexual desire [53]. This can explain why being a woman in power is not solely associated with privilege, but also, in some contexts, associated with increased vulnerability, as women in power may pose a threat to men’s power positions. It is possible that this effect differs between genders, however, gender-stratified analyses were not feasible due to the low number of men reporting both low relational justice and experiences of sexual harassment.

## Study strengths and limitations

A strength of the study is that we could control for key confounders. Furthermore, despite a generally low response rate, the large sample size allowed for exploration of potential effect modification. Moreover, validated scales were used for the outcome measure and the two main exposures. Information on potential clustering (e.g., by unit or department) was not available in the data which may have led to underestimated standard errors if within-cluster correlations were present. However, given the large sample size, the number of units in the data is likely to be substantial and observations distributed across many potential, albeit unknown, clusters, which reduces the risk of bias. A comparison between the study participants and the target population showed small differences with women, older age groups and participants with permanent employment marginally overrepresented among study participants. However, considering the aim of the study was to examine associations we do not think this is a major concern. The study is based on cross-sectional data, from which causal relationships cannot be ascertained. Although, it is generally acknowledged that organisational climate is a relevant antecedent to sexual harassment [21, 54] experiences of being sexually harassed may also influence how managers are perceived, particularly with regard to fairness and authoritarianism. In this case, perceived low relational justice and authoritarian treatment could represent consequences rather than antecedents of sexual harassment. The associations observed in this study may therefore best be understood as indicating a broader organisational context in which harassment is more likely to occur, characterised by perceived injustice and authoritarian leadership. Future longitudinal research is needed to investigate these relationships and establish their temporal ordering. Regarding the generalisability of the study results to other cultural or occupational contexts, we argue that the effect is general, based on our theoretical assumptions and supported by previous studies. However, both the national and workplace context may influence the magnitude of the effect.

## Conclusions

In conclusion, we found statistically significant associations between employee’s perceptions of workplace relational justice and authoritarian treatment and experiences of workplace sexual harassment, suggesting that superiors’ or managers unjust or authoritarian treatment of employees might be contextual factors contributing to workplace sexual harassment. Furthermore, women, employees with a foreign background or a high academic position seem to be disproportionally affected by sexual harassment under these conditions. Our study results contribute to a broader understanding of contextual factors that may facilitate workplace sexual harassment, implicating that comprehensive strategies promoting a general climate of fairness and respect, combined with intolerance of harassing behaviours, may represent a promising way forward in practice. To ensure evidence-based interventions, researchers should incorporate contextual risk factors in the design and evaluation of workplace interventions aimed at reducing harassing behaviours.

## Data Availability

The dataset analysed during the current study is not publicly available because of its sensitive nature. Data are available from Lund University (contact via the correspondent author) for researchers who meet the criteria for access to confidential data.

## Abbreviations

AT: Authoritarian treatment
CI: Confidence interval
LUSHI: Lund University Sexual Harassment Inventory
PR: Prevalence ratio
RJ: Relational justice
SH: Sexual harassment
